# Serum Metabolomic Analysis of Male Patients with Cannabis or Amphetamine Use Disorder

**DOI:** 10.3390/metabo12020179

**Published:** 2022-02-14

**Authors:** Fawaz Alasmari, Mohammed A. Assiri, Syed Rizwan Ahamad, Sahar R. Aljumayi, Wedad H. Alotaibi, Majd M. Alhamdan, Khalid Alhazzani, Metab Alharbi, Faleh Alqahtani, Abdullah F. Alasmari

**Affiliations:** 1 Department of Pharmacology and Toxicology, College of Pharmacy, King Saud University, P.O. Box 2457, Riyadh 11451, Saudi Arabia; moassiri@KSU.EDU.SA (M.A.A.); 442203305@student.ksu.edu.sa (S.R.A.); 436200767@student.ksu.edu.sa (W.H.A.); 437204095@student.ksu.edu.sa (M.M.A.); kalhazzani@KSU.EDU.SA (K.A.); mesalharbi@ksu.edu.sa (M.A.); afaleh@ksu.edu.sa (F.A.); afalasmari@KSU.EDU.SA (A.F.A.); 2 Department of Pharmaceutical Chemistry, College of Pharmacy, King Saud University, P.O. Box 2457, Riyadh 11451, Saudi Arabia; srahamad@ksu.edu.sa

**Keywords:** cannabis, amphetamine, metabolites, serum, amino acids, fatty acids, sugars

## Abstract

Studies have demonstrated that chronic consumption of abused drugs induces alterations in several proteins that regulate metabolism. For instance, methamphetamine exposure reduces glucose levels. Fatty and amino acid levels were altered in groups exposed to abused drugs. Therefore, in our study, we investigated the serum metabolomic profile of patients diagnosed with cannabis and/or amphetamine use disorders. Blood was obtained from subjects (control, amphetamine, and cannabis). Detection of serum metabolites was performed using gas chromatography. The ratio peak areas for metabolites were analyzed across the three groups. Both cannabis and amphetamine groups showed higher *d*-erythrotetrafuranose, octadecanoic acid, hexadecenoic acid, trans-9-octadecanoic acid, lactic acid and methyl thio hydantoin metabolites compared with the control group. Moreover, cannabis patients were found to possess higher glycine, 9,12 octadecanoic acid malonic acid, phosphoric acid and prostaglandin F1a than controls. Our analysis showed that the identified metabolic profile of cannabis or amphetamine use disorder patients was different than control group. Our data indicated that chronic exposure to cannabis or amphetamine dysregulated metabolites in the serum. Future studies are warranted to explore the effects of these abused drugs on the metabolic proteins.

## 1. Introduction

Amphetamine and cannabis addiction are common in many countries [1,2], including Saudi Arabia [3]. Moreover, the prevalence of drug dependence is increasing in developed countries [1,4]. It is estimated that more than 180 million people globally (approximately 4%) are using cannabis. Medical purposes have led to legalization of cannabis and amphetamine use in many countries. Amphetamine is approved for the treatment of attention deficit hyperactivity disorders. Reducing the progression of amphetamine and cannabis dependence could provide beneficial consequences clinically and economically. These two abused drugs have been involved in increasing the mortality and morbidity rates in humans [5,6,7]. Although less is known about the effects of chronic exposure to cannabis and amphetamine on the vital organs, toxicological effects have been observed [8,9,10,11,12]. Brain, lung and liver toxicity were observed in animals exposed to amphetamine [13,14,15]. In our study, we determined whether these toxicological effects, induced by cannabis or amphetamine exposure, led to dysregulation of the essential metabolites, including certain amino acids, sugars and fatty acids and other metabolites.

A prior study found that methamphetamine exposure reduced the levels of glucose in the plasma of humans [16]. Another study reported an increase in the levels of leucine in mice exposed to repeated administration of d-amphetamine [17]. These findings indicate that exposure to drugs of abuse can induce impairments in protein expressions or functions that regulate the metabolism of glucose and other metabolomics biomarkers. For instance, subcutaneous injection of nicotine (2 mg/kg) for 4 weeks has been found to induce marked changes in glucose oxidation in the cortex, sub-cortex and cerebellum of rats [18]. However, less is known about the effects of amphetamine and cannabis use disorders on the amino acids, fatty acids and sugars as well as other metabolites in the peripheral system in humans. In our study, we investigated the effects of amphetamine or cannabis use disorder, as well as cannabis–amphetamine use disorder on the essential metabolites, such as d-glucose, maltose, glycine, hydantoin, l-valine and others in the serum using gas chromatography (GC). 

It has been reported that the ions and metabolites are regulated by particular proteins, such as enzymes and transporters. Glucose, for example, is regulated by glucose transporters, and these transporters are present in different isoforms [19]. Moreover, metabolites have been found as substrates or products of several enzymes and these enzymes are present in blood cells. For instance, it is known that glycine is modulated by serine hydroxymethyl transferase [20]. Carbamoyl-phosphate synthetase 1, ornithine transcarbamylase and n-acetylglutamate synthase are essential enzymes in regulating the urea cycle [21]. Thus, the protein and gene expression levels of these proteins are involved in the modulation of the levels of these metabolic parameters in the blood.

Alternatively, studies have found a positive correlation between neuropsychiatric diseases and dysregulated human enzyme activity [22,23]. Prior studies reported that altered metabolic parameters have been found in patients who are suffering from neuropsychiatric diseases such as schizophrenia [22,23]. These enzymes are critical in regulating body growth. For example, it has been found that schizophrenia is associated with altered metabolic pathways, suggesting that the central nervous system plays a crucial role in modulating metabolite concentrations in the body [22,23]. Changes in metabolomic profiles were also observed with patients with depression [24,25], Alzheimer’s disease [26,27] and autism spectrum disorders [28,29].

Heart, lung and renal functions were dysregulated following exposure to amphetamine or cannabis [8,9,10,11,12]. For example, heart functions were altered following exposure to amphetamine [30,31]. Moreover, an association between cannabis exposure and alterations of aortic stiffness and cardiac mechanics was reported [32]. Significant alterations to liver enzymes and liver morphology were observed in subjects who chronically used marijuana [33]. Moreover, amphetamine was found to cause liver damage [34]. A case report found that amphetamine exposure was associated with hepatocellular damage [35]. 

A previous study determined the effects of psychostimulants on the metabolome profile in humans [36,37]. Serum samples were used extensively in prior metabolomics studies that investigated the effects of many diseases, such as Parkinson’s disease [38], attention deficit/hyperactivity disorder [39], amyotrophic lateral sclerosis [40] and Alzheimer’s disease [41]. In the current study, we investigated the effect of chronic exposure to cannabis or amphetamine on the metabolomics profile in the serum of addicted Saudi males. Our work provided clear insight as whether these abused drugs affect the concentrations of essential metabolites, including fatty acids, sugars, amino acids and others. 

## 2. Results 

### 2.1. Effects of Cannabis and/or Amphetamine on Metabolic Profile in Patients with Amphetamine or Cannabis Use Disorders

#### 2.1.1. Effects Cannabis or Amphetamine Use Disorder on Selected Sugars

**Figure 1:**
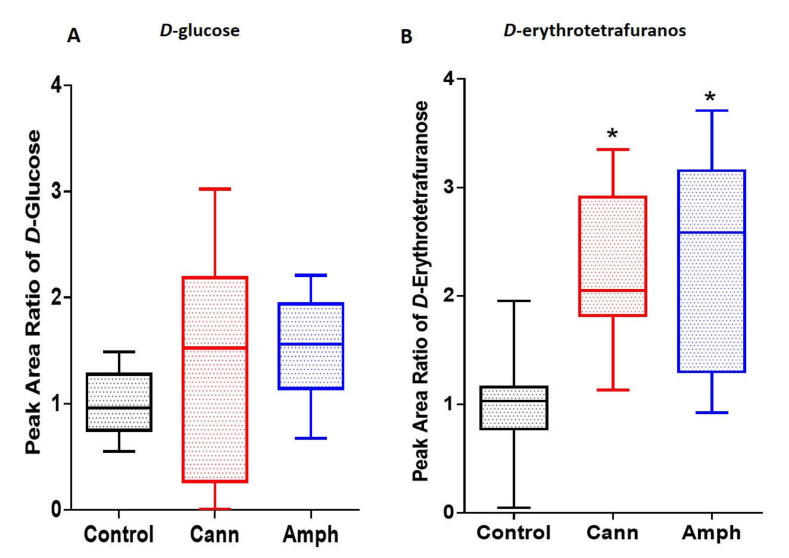
Effects of cannabis or amphetamine on selected sugars in cannabis or amphetamine use disorder patients. (**A**) Kruskal–Wallis test followed by Dunn’s multiple comparisons analysis did not reveal significant differences in *d*-glucose between groups. (**B**) Kruskal–Wallis test followed by Dunn’s post hoc analysis showed an increase in *d*-erthroterofuranose in the cannabis or amphetamine groups compared with the control group. (* *p* < 0.05). Cann, cannabis. Amph, amphetamine.

We evaluated the effects of cannabis or amphetamine on selected sugars, such as *d*-glucose and *d*-erythroterofuranose. The Kruskal–Wallis test did not show any significant differences in *d*-glucose among amphetamine, cannabis and control groups (Figure 1A). However, the analysis showed that *d*-erythrofuranose was significantly different among the groups (Figure 1B). The Dunn’s multiple comparisons tests revealed that there was a marked increase in d-erthroterofuranose in the amphetamine or cannabis groups compared with the control group.

#### 2.1.2. Effects Cannabis or Amphetamine Use Disorder on Selected Amino Acids

**Figure 2:**
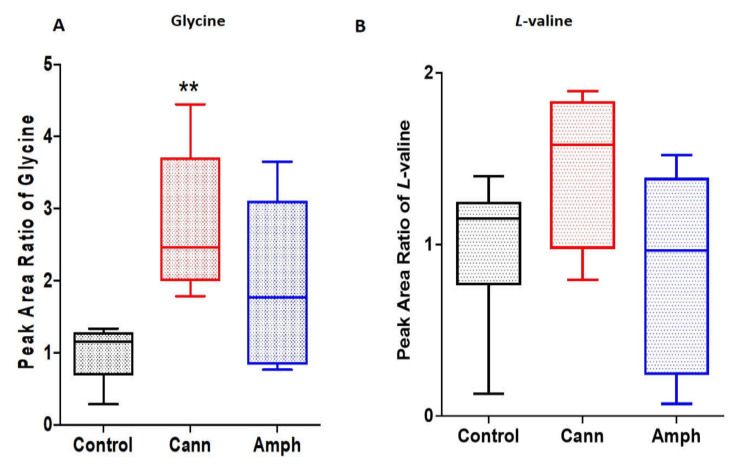
Effects of cannabis or amphetamine on selected amino acids in cannabis or amphetamine use disorder patients. (**A**) Kruskal–Wallis test followed by Dunn’s post hoc analysis showed a significant increase on glycine in the cannabis compared with the control group. (**B**) Kruskal–Wallis test followed by Dunn’s post hoc analysis did not show significant differences in *l*-valine between the three groups. (** *p* < 0.01). Cann, cannabis. Amph, amphetamine.

We further determined the effects of cannabis and/or amphetamine on selected amino acids, such as glycine and *l*-valine. Using the Kruskal–Wallis test, the analysis showed significant changes to glycine among the groups (Figure 2A). Dunn’s multiple comparisons analysis showed a significant increase in glycine in cannabis group compared with the control group. Although the Kruskal–Wallis test showed significant differences in *l*-valine metabolite among the groups (Figure 2B), the post hoc test did not show differences between groups.

#### 2.1.3. Effects of Cannabis or Amphetamine Use Disorder on Selected Fatty Acids

**Figure 3:**
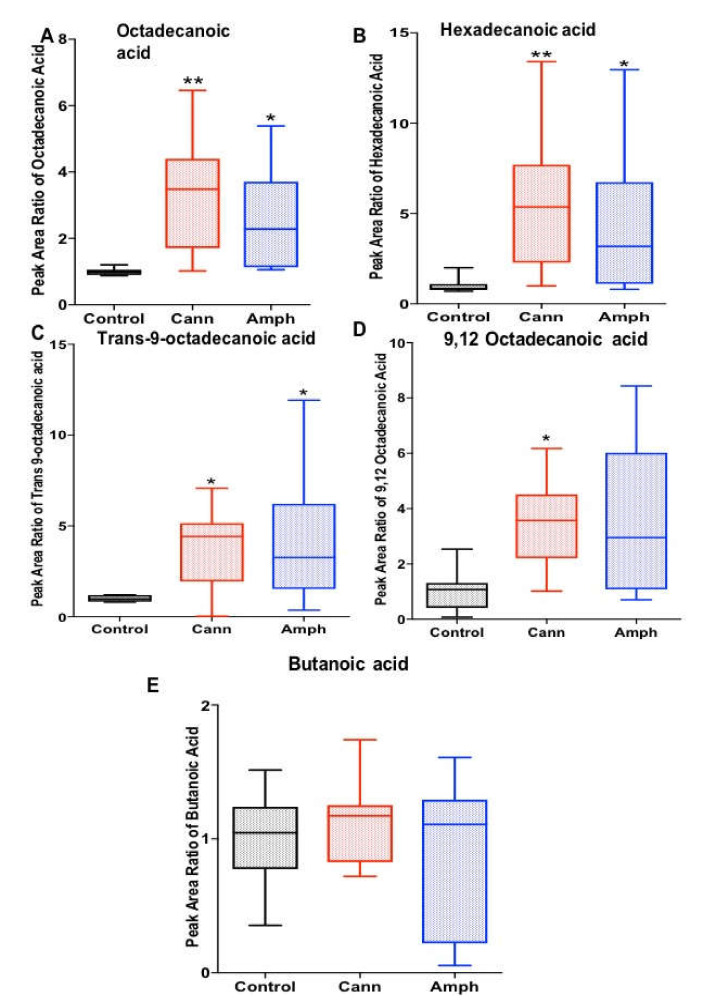
Effects of cannabis or amphetamine on selected fatty acids in cannabis or amphetamine use disorder patients. (**A**) Kruskal–Wallis test followed by Dunn’s post hoc analysis showed amphetamine and cannabis groups possessed higher octadecanoic acid compared with control group. (**B**) Kruskal–Wallis test followed by Dunn’s post hoc analysis showed amphetamine and cannabis groups possessed higher hexadecenoic acid compared with control group. (**C**) Kruskal–Wallis test followed by Dunn’s post hoc analysis showed that *trans*-9-octadecanoic acid was higher in the amphetamine or cannabis group compared with the control group. (**D**) Kruskal–Wallis test followed by Dunn’s post hoc analysis revealed that 9,12 octadecanoic acid was higher in the cannabis group compared with the control group. (**E**) Kruskal–Wallis test followed by Dunn’s post hoc analysis did not reveal significant changes in butanoic acid between the groups. (* *p* < 0.05, ** *p* < 0.01). Cann, cannabis. Amph, amphetamine.

We investigated the effects of cannabis and/or amphetamine on selected fatty acids, including butanoic acid, octadecanoic acid, hexadecenoic acid and *trans*-9-octadecanoic acid. The Kruskal–Wallis test showed significant differences in octadecanoic acid (Figure 3A), hexadecenoic acid (Figure 3B), *trans*-9-octadecanoic acid (Figure 3C) and 9,12 octadecanoic acid (Figure 3D) among the groups. However, the statistical analysis did not reveal any significant changes in butanoic acid among the groups (Figure 3E). Dunn’s multiple comparisons analysis showed that the amphetamine and cannabis groups possessed higher oxadecanoic acid, hexadecenoic acid and *trans*-9-octadecanoic acid compared with the control group. Dunn’s multiple comparisons analysis showed that the cannabis group possessed higher 9,12 octadecanoic acid compared with the control group. 

#### 2.1.4. Effects of Cannabis or Amphetamine Use Disorder on Malonic Acid, Lactic Acid, and Phosphoric Acid

**Figure 4:**
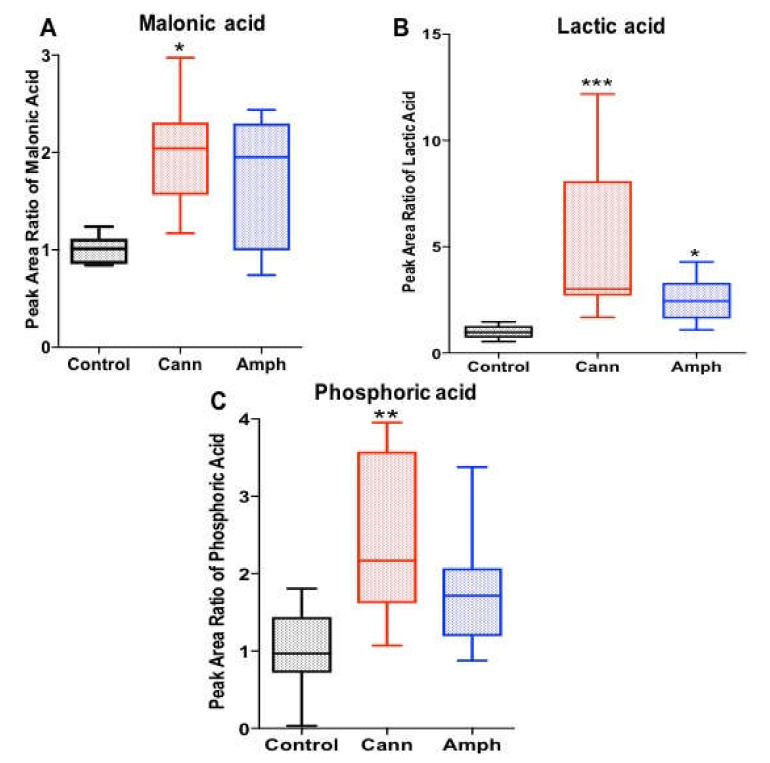
Effects of cannabis or amphetamine on malonic acid, lactic acid and phosphoric acid in cannabis or amphetamine use disorder patients. (**A**) Kruskal–Wallis test followed by Dunn’s post hoc analysis revealed that the cannabis group possessed higher malonic acid metabolite compared with the control group. (**B**) Kruskal–Wallis test followed by Dunn’s post hoc analysis revealed that both cannabis and amphetamine groups possessed higher lactic acid metabolite compared with the control group. (**C**) Kruskal–Wallis test followed by Dunn’s post hoc analysis revealed that the cannabis group possessed higher phosphoric acid metabolite compared with the control group. (* *p* < 0.05, ** *p* < 0.01, *** *p* < 0.001). Cann, cannabis. Amph, amphetamine.

The Kruskal–Wallis tests showed significant differences in malonic acid (Figure 4A), lactic acid (Figure 4B) and phosphoric acid (Figure 4C) metabolites among the groups. Using Dunn’s multiple comparisons test, the analysis showed that the cannabis group had higher malonic acid and phosphoric acid compared with controls. Moreover, the analysis showed that lactic acid was higher in both cannabis and amphetamine groups compared with the control group. 

#### 2.1.5. Effects of Cannabis or Amphetamine Use Disorder on Hydantoin and Methyl’ib Thio Hydantoin

**Figure 5:**
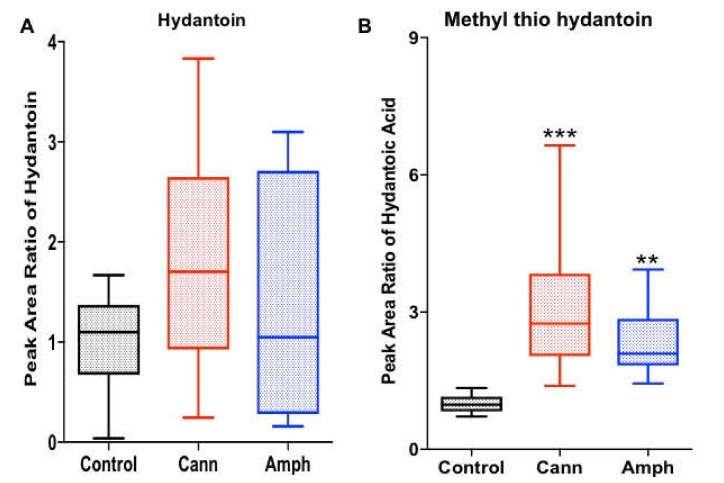
Effects of cannabis or amphetamine on hydantoin and methyl thio hydantoin in cannabis or amphetamine use disorder patients. (**A**) Kruskal–Wallis test followed by Dunn’s post hoc analysis did not show any differences in hydantoin among the groups. (**B**) Kruskal–Wallis test followed by Dunn’s post hoc analysis showed that methyl thio hydantoin was higher in both amphetamine and cannabis groups compared with the control group. (** *p* < 0.01, *** *p* < 0.001). Cann, cannabis. Amph, amphetamine.

We further investigated the effects of cannabis or amphetamine on hydantoin and methyl thio hydantoin. Although the statistical analysis did not show any significant differences in hyndantoin among all four groups (Figure 5A); methyl thio hydantoin was differentially changed among the groups (Figure 5B). Dunn’s multiple comparisons test showed that methyl thio hydantoin was increased in both amphetamine and cannabis groups compared with the control group. 

#### 2.1.6. Effects of Cannabis or Amphetamine Use Disorder on Other Metabolites

**Figure 6:**
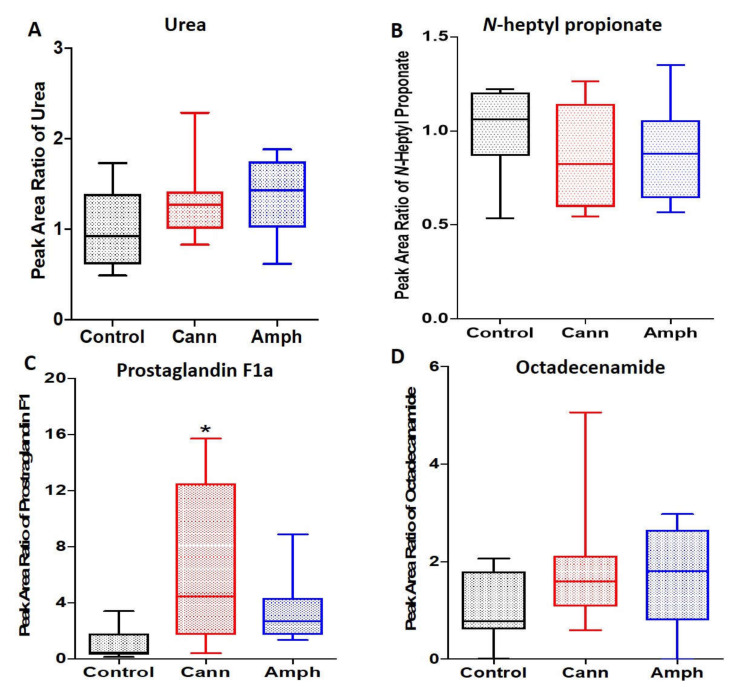
Effects of cannabis or amphetamine on other metabolites in cannabis or amphetamine use disorder patients. (**A**) Kruskal–Wallis test followed by Dunn’s post hoc analysis did not reveal any significant changes in urea between groups. (**B**) Kruskal–Wallis test followed by Dunn’s post hoc analysis did not reveal any significant changes in *n*-heptyl propionate between groups. (**C**) Kruskal–Wallis test followed by Dunn’s post hoc analysis showed that prostaglandin F1a was higher in cannabis group compared with control group. (**D**) Kruskal–Wallis test followed by Dunn’s post hoc analysis did not reveal any significant changes in octadecenamide between groups. (* *p* < 0.05). Cann, cannabis. Amph, amphetamine.

We determined the effects of amphetamine or cannabis use disorder on urea, *n*-heptylpropionate, prostaglandin F1a and 9-octadecenamide. The Kruskal–Wallis tests showed non-significant differences in urea (Figure 6A), *n*-heptylpropionate (Figure 6B) and octadecenamide (Figure 6D) among the groups. The Kruskal–Wallis tests showed significant changes in prostaglandin F1a among the groups (Figure 6C). Dunn’s multiple comparisons analysis revealed that prostaglandin F1a was higher in cannabis group as compared with the control group. 

### 2.2. Metabolomic Profiles Analysis

#### 2.2.1. Partial Least Squares Discriminant Analysis (PLS-DA)

**Figure 7:**
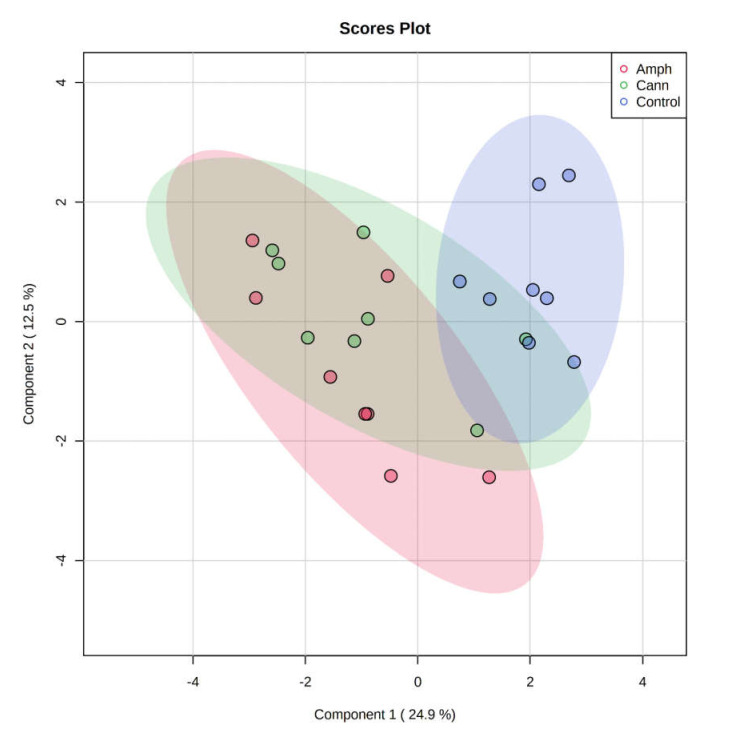
Metabolomic profiles analysis. Partial least squares discriminant analysis of the metabolomic data. Purple dots represent individuals in the control group; Green dots represent individuals in the cannabis group; Red dots represent individuals in the amphetamine group. Cann, cannabis. Amph, amphetamine.

To visualize the distribution and differences of the metabolites between the groups, we employed partial least squares discriminant analysis (PLS-DA) model. The PLS-DA revealed that there is a separation between the samples of amphetamine or cannabis patients from control group (Figure 7). 

#### 2.2.2. Metabolites Analysis 

**Figure 8:**
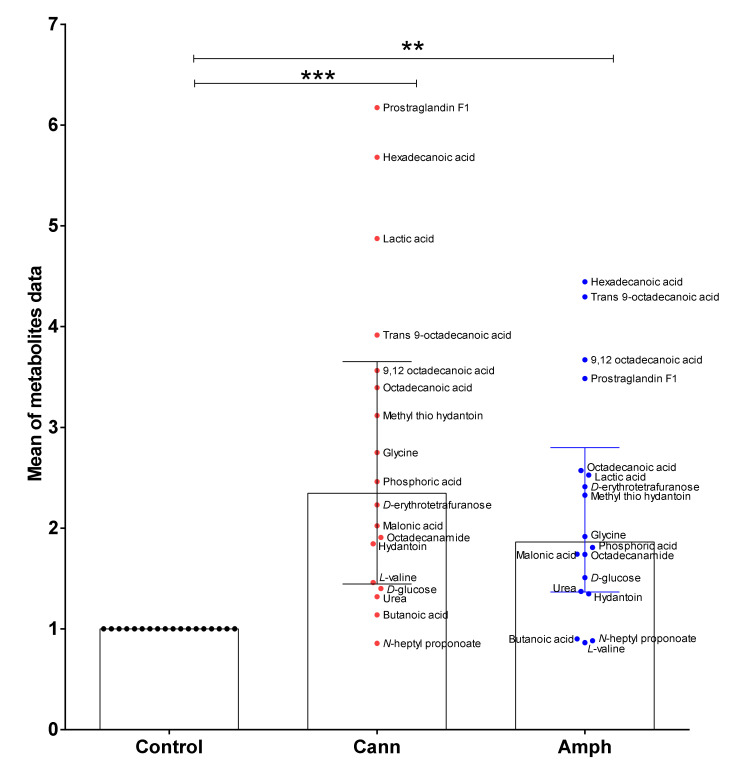
Kruskal–Wallis test followed by Dunn’s post hoc analysis showed that the metabolomic profiles of amphetamine or cannabis use disorder patients are significantly different than control group. Data are expressed as median with interquartile range. (** *p* < 0.01, *** *p* < 0.001). Each dot represents mean of one metabolite in each group. Cann, cannabis. Amph, amphetamine.

Moreover, the mean data of all metabolites were analyzed as one parameter between the three groups. Kruskal–Wallis test followed by Dunn’s post hoc analysis showed that the metabolomic profiles of amphetamine or cannabis use disorder patients are significantly different than the control group (Figure 8). 

## 3. Discussion

Emerging evidence suggests that the exposure to cannabis or amphetamine is becoming popular worldwide [1,2]. Thus, finding molecular therapy targets is important to reduce the toxicological effects that can occur from exposure to amphetamine or cannabis. In our study, we determined whether these toxicological effects led to dysregulation in the levels of metabolic biomarkers in the blood of subjects that were exposed to cannabis or amphetamine. We found that cannabis or amphetamine induced a marked increase in *d*-erythrotetrafuranose and methy thio hydantoin. Additionally, we found here that cannabis could increase lactic and malonic acids, while both cannabis and amphetamine increased lactic acid in the blood. Moreover, both amphetamine and cannabis showed an ability to increase hexadecanoic acid, octadecanoic acid and *trans*-9-octadecanoic acid compared with the control group. In addition, cannabis was able to increase 9,12 octadecanoic acid, prostaglandin F1a and glycine in blood of cannabis use disorder patients. These metabolic parameters were assessed by measuring the peak areas using the GC technique.

Studies have reported that fasting serum glucose level was reduced in methamphetamine, an amphetamine analog, abusers in China [16]. A study reported that methamphetamine increased the release of insulin in pre-clinical models [42]. Therefore, methamphetamine might decrease the level of glucose through increasing insulin secretion and actions. Moreover, a prior work demonstrated that blood sugar level was decreased in non-diabetic and diabetic individuals exposed to 5–10 g of cannabis daily for one year compared with control group [43]. Although we did not observe any differences in glucose level in both amphetamine and cannabis groups as compared to control group, there is a trend of increase in both diseased groups compared with controls. Moreover, cannabis and amphetamine-exposed groups showed higher *d*-erythrofuranose as compared to the control group. This might indicate that cannabis and amphetamine affect sugar pathways, particularly the furanose pathway. However, this hypothesis needs further elucidation. Future research should determine the effects of amphetamine and cannabis on sugars pathways, including lactose degradation and synthesis, gluconeogenesis and glycolysis pathways. The molecular research is important to specifically identify the pathway involved in the effects of amphetamine and cannabis on the serum sugars levels.

Our metabolomic data also suggest that cannabis use disorders might be associated with altered carnitine synthesis pathways. We found here that glycine was increased in the cannabis group as compared to the control group. Glycine is involved in the carnitine synthesis pathway [44,45]. Moreover, glutamate metabolism involves the glycine pathway [46]. Investigating the effects of cannabis constituents on the enzymes that regulate glycine can be a novel approach to further determine the responsible signaling pathways.

Our findings indicated that malonic and phosphoric acids were increased in the cannabis group, while lactic acid was higher in both amphetamine and cannabis groups compared with the control group. Moreover, the amphetamine group showed a non-significant high phosphoric acid level. Phosphoric acid is essential to support the teeth, bones and kidneys [47,48,49]. Imbalance of phosphoric acid levels might lead to negative consequences. For example, cannabis smoke and amphetamine exposure might lead to negative effects on teeth and bone mass, respectively [50,51]. Malonic acid plays a key role in the biosynthesis of fatty acids [52,53]. Lactic acid is an essential metabolite involved in gluconeogenesis and pyruvate metabolism [54,55]. Further studies are warranted to explore the specific effects of cannabis or amphetamine on regulatory proteins level of serum acids metabolites.

Although hydantoin was not changed in amphetamine nor cannabis use disorder patients, both groups exhibited higher methyl thio hydantoin as compared to the control group. This indicates that cannabis and amphetamine modulate methyl and thio functional groups transfer. We further determined the effects of amphetamine or cannabis on certain fatty acids. We reported that octadecanoic acid, hexadecanoic acid and *trans*-9-octadecanoic acid were higher in both amphetamine and cannabis groups, indicating that amphetamine and cannabis modulate fatty acid metabolism. Plasma pools of free fatty acids were increased in rats exposed to amphetamine, probably through increasing fatty acids production [56]. Cannabis plants also contain fatty acids [57,58]. However, butanoic acid was not changed in cannabis or amphetamine groups compared with the control group. We also observed a non-significant high level of prostaglandin F1 in the amphetamine group, but a significant high level with cannabis group, compared with the control group. The trend changes in this metabolite indicate that amphetamine or cannabis may modulate the pain and inflammatory pathways. Although cannabinoids might have anti-inflammatory properties [59], uncontrolled amounts of cannabinoid and non-cannabinoid exposure and duration may produce different effects. Moreover, chronic amphetamine exposure was associated with increased blood interleukine-4 levels [60]. The alterations in sugars, amino acids and fatty acids in the amphetamine or cannabis groups might be associated with changes in signaling pathways involved with these altered metabolites in the peripheral system.

Studies show positive correlations between exposure to abused drugs and other diseases/conditions [61,62,63]. These conditions are associated with dysregulated metabolomics pathways. The inflammatory and immunological regulatory receptors, transporters and enzymes have been found to be involved in modulating the levels of biological biomarkers, including metabolic biomarkers in the blood [64,65,66]. Cardiovascular diseases are also associated with changes in the serum levels of ions and metabolites, which may further progress the diseases. For instance, it has been found that there are changes in the serum levels of amino acids, acetylcarnitine, carnitine, and phosphatidylcholines in patients who have heart failure [67]. The changes in the activities or expression of the enzymes that regulate the levels of the amino acids, sugars and fatty acids after exposure to cannabis or amphetamine may be the mechanism for the dysregulatory effects of these abused drugs on the essential metabolites in the blood; this was investigated in our study.

## 4. Materials and Methods

### 4.1. Ethical Considerations and Informed Consent

The proposed study was approved by the Institutional Review Board (IRB) committee of College of Medicine, King Saud University, Riyadh, Saudi Arabia (20/0212/IRB) and Eradah Complex for Mental Health (H-01-R-063-10). The research study is in accordance with the guidelines of the IRB Committee. The study was carried out following the rules of the Declaration of Helsinki of 1975 and later amendments. Patients agreed with the objective of our study and signed the consent forms.

### 4.2. Study Design and Blood Collection

In our study, 24 patients, enrolled in our study, were divided into three groups; Group 1 was the control group (8 individuals); Group 2 was the cannabis use disorder group (8 patients); Group 3 was the amphetamine use disorder group (8 patients). The Diagnostic and Statistical Manual of Mental Disorders (DSM–5) criteria were used for diagnosis of amphetamine or cannabis use disorder. Clinical and demographic information are highlighted in Table 1. Blood samples were obtained at the Eradah Complex for Mental Health. Peripheral blood samples were collected by venipuncture and serum separator tubes at the time where amphetamine and/or cannabinoids were detected in the blood. All participants were free from medical history of obesity, psychotic disorders, renal diseases, blood disorders, or other infectious diseases. Participants declared no medical history of diabetes. Although food intake plays a role in modulating metabolites, we collected the blood samples at the same time of day, which was early in the morning, before breakfast, for all four groups.

### 4.3. Gas Chromatography Mass Spectroscopy (GC-MS)

#### 4.3.1. Chemicals

The following chemicals were bought from Sigma-Aldrich, St. Louis, MO, USA; pyridine, bis-*N*,*O*-trimethylsilyl trifluoroacetamide (BSTFA) methoxyamine hydrochloride, and chlorotrimethylsilane (TMCS). Methanol and hexane were bought from BDH VWR international ltd Poole, BH 15 1TD, England. Milli-RO and Milli-Q Plus instruments (Millipore, Billerica, MA, USA) were used to obtain deionized water.

#### 4.3.2. Derivatization 

Serum samples were thawed at room temperature and vortexed for 2 min. A 100 µL serum sample was pipetted into a tube and mixed with 100 µL of MilliQ water and 400 µL of methanol. The tube was vortexed at room temperature for 2 min and centrifuged at 15,000× *g* for 10 min at 4 °C. A 200 µL sample was pipetted from the supernatant of each sample and transferred into a GCMS vial. The solution was rinsed with *nitrogen* air to dry the vials completely. Next, 100 µL of methoxyamine hydrochloride was added into a pyridine solution (15 mg mL^−1^). This mixture was vortexed for 10 min and the sample was stored at room temperature for 16 h. Next, a derivatization process was performed into the methoxymate sample using 100 µL BSTFA/TMCS (99/1, *v/v*), vortexed again for 10 min, and stored at 50 °C for 2 h. A split mode injection system was used and 1 µL of the derivatized sample was injected (split ratio 1:20) [68,69].

#### 4.3.3. Parameter Description

Gas chromatography–mass spectrometry (GC-MS) analysis of the serum samples was performed using a PerkinElmer Clarus 600 gas chromatograph, a Clarus 600 T mass spectrometer and the Turbomass software, as described in prior work [68,69]. A GCMS system equipped with an elite 5MS column with a length of 30.0 m, an inner diameter of 0.25 mm and a thickness of 0.5 µm (PerkinElmer, Waltham, MA, USA) was used to take an aliquot (1 μL) of the derivatized sample. The stationary phase used here was fused silica, while the mobile phase was He gas; the mobile phase was delivered at flow rate of 1.0 mL/min. The injector temperature was 280 °C; the GC–MS system starting oven temperature was 40 °C and was increased to 200 °C with a heating rate of 5 °C, then increased again to 300 °C with the same heating rate. The total run time was 32 min. The injector and interface temperatures were controlled to 220 °C and 240 °C, respectively. At scanning of 50–600 *m/z*, the electron ionization mode was used for the MS detection. The electron energy and multiplier voltage were maintained at 70 eV and 330 V, respectively. Due to pyridine and the derivatizing agent, the solvent delay was 6 min. Using the National Institute of Standards and Technology (NIST) library (2005) and the Wiley library (2006), unknown compounds were determined through spectra comparisons. The determined metabolites and matched retention time are shown in Table 2. 

### 4.4. Statistical Analysis

GraphPad prism software was used to analyze the metabolomics profiles of control and patients diagnosed with cannabis or amphetamine use disorder. Non-parametric Kruskal–Wallis tests were used to determine the significant of metabolite peak areas ratio among groups. The ratio peak areas were calculated through dividing each peak area by the average peak area of control group for each metabolite. Dunn’s multiple comparisons tests were used to study whether there were any significant changes in metabolite peak area ratios between groups. 

### 4.5. PLS-Discriminant Analysis Model

PLS-DA model was created to predict the distribution and classification of the metabolomics biomarkers differences between the studied groups using MetaboAnalyst program.

## 5. Conclusions

Our current work sheds light on specific metabolites altered in patients with cannabis or amphetamine disorder. The focus of future research work should be on investigating the molecular pathways regulating the levels of these altered metabolites in the serum. In addition, restoring the changes in the serum metabolites levels in patients with amphetamine or cannabis disorder might support their health and body development. Our work provides a clear understanding of the possible involvement of these changed metabolites on synthesis and metabolism of amino acids, sugars, fatty acids and other metabolite groups. However, the present work should be validated using a larger sample size. Our current work opens new ways for upcoming research work to further explore the proposed signaling pathways. In addition, our present study was conducted only on male patients; future metabolomic work may perform the same analysis on female and male patients with a larger sample size to study whether there are any gender differences on the metabolomic profiles of amphetamine or cannabis use disorder patients. Future research studies are also warranted to quantify the concentrations of the identified metabolites in patients with amphetamine or cannabis use disorder.

## Figures and Tables

**Table 1:** Clinical and demographic information of participants enrolled in the study. Age is expressed as mean ± SD. Cann, cannabis. Amph, amphetamine. HIV, human immunodeficiency virus. HPC, hepatitis C virus. TB, tuberculosis. F, female. M, male.

	Control Group	Cann Group	Amph Group
No. of patients	8	8	8
Gender	8M, 0F	8M, 0F	8M, 0F
Age (Mean ± SD)	30.38 ± 4.37	27.88 ± 6.83	31 ± 6.55
Marital status	3 married, 5 single	2 married, 6 single	2 married, 6 single
Infectious diseases (HIV, HCV, TB)	Negative	Negative	Negative
Substance dosage use	None	Smoking	Oral tablets
Substance use history 1–4 years 5–9 years 10–14 years ≥15 Years	None None None None	1 patient 3 patient 2 patients 2 patients	1 patient 3 patients 2 patients 2 patients

**Table 2:** Identified metabolites and their retention time using the gas chromatography technique. RT, retention time.

#	Metabolites	RT
1	Malonic acid	6.21
2	*L*-Valine	7.34
3	Butanoic acid	8.21
4	Hydantoin	8.51
5	Lactic acid	9.04
6	*N*-Heptyl propanoate	9.50
7	Methyl thio hydantoin	11.45
8	Urea	11.90
9	Phosphoric acid	12.34
10	Glycine	12.68
11	*D*-Erythrotetrofuranose	20.79
12	*D*-Glucose	21.26
13	Hexadecanoic acid	22.87
14	*TRANS*-9-Octadecenoic acid	24.45
15	Octadecanoic acid	24.67
16	9,12-Octadecadienoic acid	25.24
17	Octadecenamide	25.92
18	Prostaglandin F1a	28.67

## Data Availability

The data presented in this study are available within the article. Patients’ information are restricted due to privacy and ethical considerations.
